# Synthesis, FTIR, ^13^C-NMR and Temperature-Dependent ^1^H­NMR Characteristics of Bis-naphthalimide Derivatives

**DOI:** 10.3390/molecules171012427

**Published:** 2012-10-22

**Authors:** Waldemar Grzesiak, Bogumił Brycki

**Affiliations:** Laboratory of Microbiocides Chemistry, Department of Chemistry, Adam Mickiewicz University, Grunwaldzka 6, 60-780 Poznań, Poland

**Keywords:** bis-naphthalimide, ^1^H-NMR spectroscopy, FTIR spectra, anticancer activity, DNA, intercalator

## Abstract

Chemotherapy is still the most important method of cancer treatment. To make this method more effective and safe, new drugs to destroy cancer cells are needed. Some bis-naphthalimide derivatives show potential anticancer activity *via* an intercalation mechanism. A higher degree of DNA intercalation corresponds to better therapeutic effects. The degree of intercalation of naphthalimides depends on their structure, molecular dynamics and intermolecular interactions with DNA. In order to apply any active substance as a drug, its molecular dynamics as well as possible interactions with target molecules have to be examined in exhaustive details. This paper describes a practical preparation of some novel bis-naphthalimide derivatives with different functional groups and their FTIR and ^1^H- and ^13^C-NMR spectral characteristics. To determine the molecular dynamics of the obtained compounds the temperature, their ^1^H-NMR spectra were measured. It has been clearly proven in this paper that the unusual temperature-dependent ^1^H-NMR behavior of the aromatic protons of phthalimide derivatives, previously described in the literature as “hypersensitivity” and explained by n-π interactions and molecular motions of aromatic amide rings, is a result of temperature driven changes of the geometry of carbonyl groups.

## 1. Introduction

1,8-Naphthalimide derivatives are a very important class of compounds. Their photophysical properties are useful in many applications as organic light-emitting diodes, [1] fluorescent dyes for solar energy collectors, [2] laser active media, [3] potential photosensitive biologically active units, [4] liquid-crystal additives, [5] effective, non-oxygen based photochemical inactivators of enveloped viruses, including herpes simplex virus and HIV [6], and as fluorescent dyes for synthetic polymers and textile materials [7,8].

The biological activity of *N*-substituted naphthalimides has been studied since the early 1940s. The activity of simple analogues includes antiviral [9], antibacterial [10] and antitrypanosomal properties [11]. Special attention has been devoted to the high anticancer activity of naphthalimides [12,13,14,15,16,17,18,19,20,21,22,23,24,25,26,27,28,29,30,31,32,33,34], which is due to their interactions with DNA by intercalation. Intercalators are molecules which consist of flat π-deficient aromatic or heteroaromatic systems that bind reversibly to double-helical DNA distorting the DNA backbone conformation and interfering with DNA-protein interactions [12,13,14]. The complex molecular structure of DNA can be influenced by intercalators through π-π, electrostatic, hydrogen bonding, dipole-dipole and hydrophobic interactions. In general the stronger the interactions of the intercalator with DNA, the higher the antitumor activity of the drug will be. In order to use any active substance as a drug, the recognition of all possible interactions with target molecules is essential. Neglecting this can lead to tragic results as in case of thalidomide*.* Because bis-naphthalimides act as DNA intercalators, therefore knowledge about the molecular dynamics of naphthalimide rings is absolutely necessary. In the case of bis-naphthalimide derivatives the problem remains unsolved to date.

Some years ago Barrett *et al*. [35] and Howell *et al*. [36] observed an unusual temperature behavior of the aromatic protons of phthalimide derivatives in ^1^H-NMR. In certain temperature ranges only one peak was observed for four different aromatic protons. Authors explained it as “hypersensitivity” caused by n-π interactions of the n-electrons of substituents with the π-electrons of aromatic rings together with the molecular dynamics of these phthalimide systems. They also calculated some thermodynamic parameters of these interactions.

Because this type of effect observed by Barrett [35] and Howell [36] could be very important for the interactions of bis-naphthalimides with DNA, we have also checked for it in synthesized bis-naphthalimides. We observed a similar temperature behavior for all studied bis-naphthalimides. However, the explanation is quite different from that given by Barrett. In our paper we have very proven precisely, without any doubts, that no n-π interactions are present in these systems and that the unusual behavior of protons in ^1^H-NMR spectra is caused by temperature-driven changes in the carbonyl group geometry. 

## 2. Results and Discussion

### 2.1. Synthesis

**Scheme 1:**
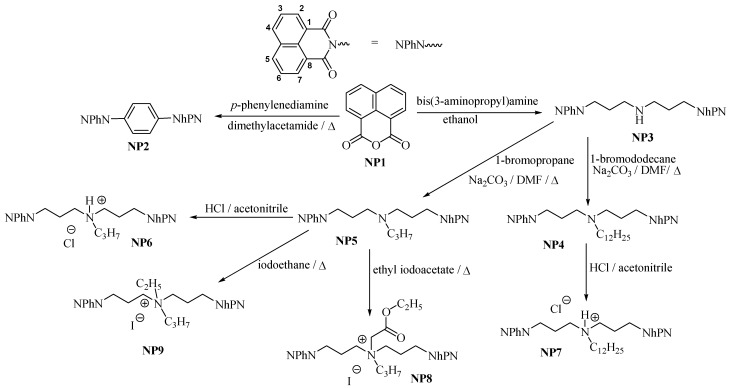
Synthesis of bis-naphthalimide derivatives.

In this paper we report the synthesis of bis-naphthalimide derivatives, their FTIR and ^13^C-NMR spectra and the unusual temperature behavior of their protons in ^1^H-NMR. The substrate used to obtain the reported bis-naphthalimide derivatives (Scheme 1) is 1,8-naphthalenedicarboxylic anhydride (**NP1**).

Reactions of anhydride **NP1** with bis-*N,N*-(3-aminopropyl)amine and *p*-phenylenediamine led to *N,N*-bis-[3-(1,8-naphthalimide)propyl]amine (**NP3**) and *N,N′*-(1,4-phenylene)-bis(1,8-naphthalimide) (**NP2**), respectively. Compound **NP2** has six π-electrons more in comparison to the other naphthalimides. Thus, its π-π interaction potency with the heteroaromatic bases in DNA is significantly enhanced. The functionalization of the spacer by nitrogen atom gives the product **NP3**, which can additionally interact with DNA by hydrogen bonding. To obtain *N,N*-bis-[3-(1,8-naphthalimido)-propyl]-*N*-propylamine (**NP5**) and *N,N*-bis-[3-(1,8-naphthalimido)propyl]-*N*-dodecylamine (**NP4**) the alkylations with alkyl chlorides, bromides or iodides in the presence of inorganic carbonates in chloroform, dichloromethane or aliphatic alcohols were carried out. The yields of these reactions were below 10%, due to the low boiling points of the solvents used. The use of high boiling solvents like dimethylformamide or dimethylacetamide leads to *N,N*-bis-[3-(1,8-naphthalimido)propyl]-*N*-propylamine (**NP5**) and *N,N*-bis-[3-(1,8-naphthalimido)-propyl]-*N*-dodecylamine (**NP4**) in yields over 75%. Moreover, the products can be easily isolated from these solvents. Compounds **NP4** and **NP5** can mainly interact with DNA by π-π interactions and hydrogen bonding. Due to the presence of hydrocarbon chains these compounds can also interact with DNA by hydrophobic interactions. The naphthalimide derivatives **NP5** and **NP4** are barely soluble in water and alcohols. To improve their solubilities in water, hydrochlorides **NP6** and **NP7** were obtained by addition of hydrochloric acid to acetonitrile solutions of **NP5** and **NP4**, respectively. The nitrogen atom in the hydrochlorides of bis-naphthalimide derivatives **NP6** and **NP7** possesses a positive charge, so additional electrostatic interactions can influence DNA structure. The quaternization of *N,N*-bis-[3-(1,8-naphthalimido)-propyl]-*N*-propylamine (**NP5**) by ethyl iodide without solvent gives *N*-ethyl-*N,N*-bis-[3-(1,8-naphthalimido)propyl]-*N*-propylammonium iodide (**NP9**), whereas the quaternization of **NP5** by ethyl iodoacetate in acetonitrile as solvent gives *N*-(2-etoxy-2-oxoethyl)-*N,N*-bis-[3-(1,8-naphthalimido)-propyl]-*N*-propylammonium iodide (**NP8**). Like the hydrochlorides **NP6** and **NP7**, quaternized compounds **NP8** and **NP9** have positively charged nitrogen atoms and electrostatic interactions with DNA can play important role in the intercalation mechanisms. Solubility in water of the synthesized bis-naphthalimide intercalators is poor; however, using gemini surfactants with high HLB values as solubilizers, satisfactory results were obtained. The synthesized bis-naphthalimide intercalators will be tested as anticancer agents.

### 2.2. FTIR Spectra Study

**Figure 1:**
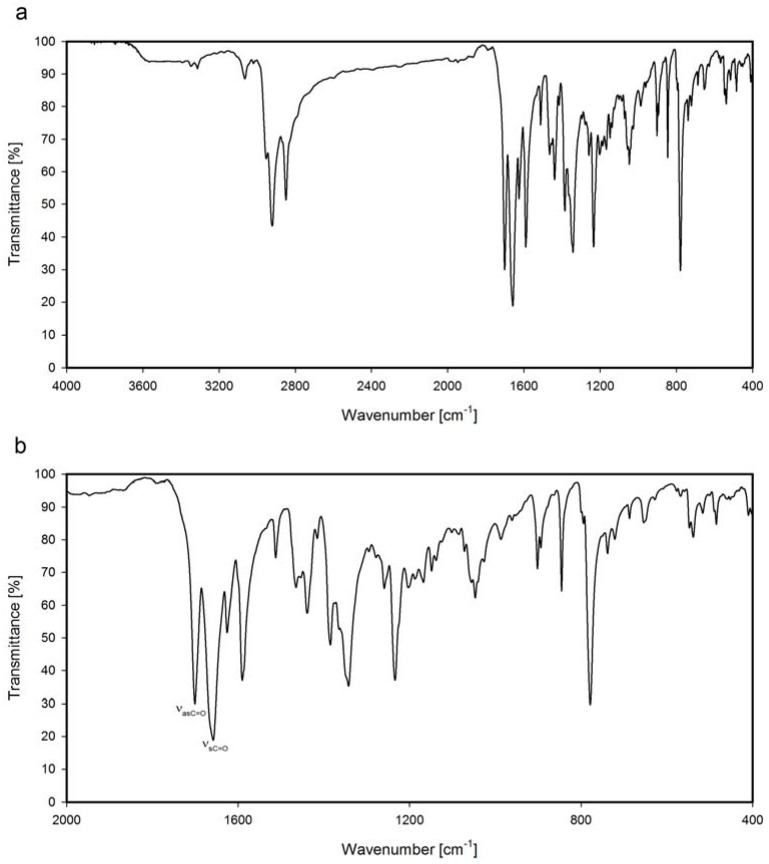
FTIR spectrum of *N,N*-bis[3-(1,8-naphthalimide)propyl]-*N*-dodecylamine (**NP4**); (**a**) range 4000–400 cm^−1^; (**b**) range 2000–400 cm^−1^.

FTIR spectra of naphthalimide derivatives **NP2**–**NP9** were recorded in KBr pellets at 298 K. The basic FTIR features of the naphthalimide structure are C-C, C-N and C=O bands. The C-C stretching vibration bands of naphthalimide ring appear in the 1630–1400 cm^−1^ range, while the C-N stretching vibration bands in the dicarboximide ring are present between 1100 and 1050 cm^−1^. In the 3100–3000 cm^−1^ region typical C-H stretching vibration bands of aromatic rings are observed, while the C-H asymmetric and symmetric stretching vibration bands of the methylene and methyl groups of *N*-substituents lie in 2960–2850 cm^−1^ the region (Figure 1).

The most characteristic bands in the FTIR spectra are the carbonyl bands. Asymmetric stretching vibration bands of the carbonyl groups of the investigated naphthalimide derivatives, ν_as_C=O, appear in the 1702–1695 cm^−1^ range, while the symmetric stretching vibration bands of carbonyl groups, ν_s_C=O, are observed between 1659–1653 cm^−1^ (Table 1).

**Table 1:** Selected FTIR frequencies for naphthalimide derivatives.

Compound	ν_asC=O_	ν_sC=O_	Others bands
	[cm^−1^]	[cm^−1^]	[cm^−1^]
NP2	1710	1681, 1667	
NP3	1695	1659	3313 ^a^
NP4	1702	1659	
NP5	1701	1656	
NP6	1698	1656	2600 ^b^
NP7	1698	1659	2459 ^c^
NP8	1696	1654	1741 ^d^
NP9	1701	1653	

^a^ *ν_N-H_* band; ^b^ *ν_N-H_*…_Cl_^−^ band; ^c^ *ν_N-H_*…_Cl_^−^ band; ^d^ *ν_esterC=O_* band.

**Figure 2:**
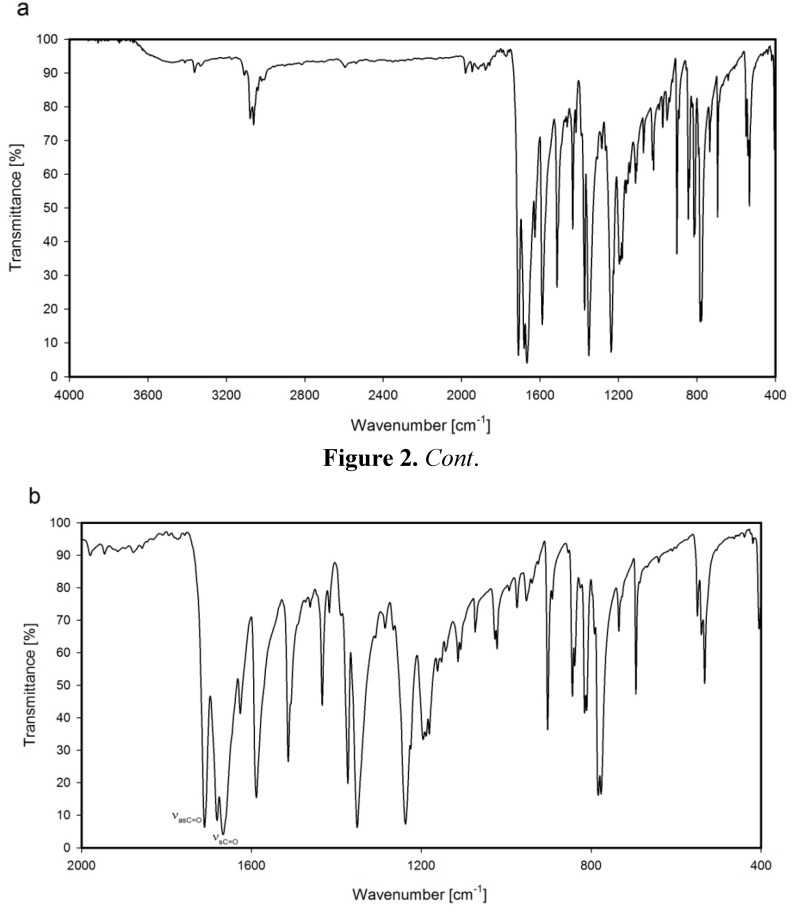
FTIR spectrum of *N,N'*-(1,4-phenylene)-bis(1,8-naphthalimide) (**NP2**); (**a**) range 4000–400 cm^−1^; (**b**) range 2000–400 cm^−1^.

The symmetric stretching vibration bands are very intense, whereas the asymmetric stretching vibration bands are much less intense. The difference of intensities is related to the symmetry of the molecule. When both carbonyl groups of dicarboximide ring are not planar, then the intensity of asymmetric vibrations is lower in comparison to the intensity of symmetric stretching vibration bands [37,38,39]. For *N,N'*-(1,4-phenylene)-bis(1,8-naphthalimide) (**NP2**) the asymmetric stretching vibration bands of the carbonyl group (ν_as_C=O), as well as symmetric stretching vibrations of the carbonyl group (ν_s_C=O) are shifted over 10 cm^−1^ to upper frequencies in comparison to the average frequencies of the others investigated naphthalimide derivatives (Table 1). Moreover, the symmetric stretching vibration of the carbonyl group in this compound is split, and two bands are observed at 1681 and 1667 cm^−1^ (Figure 2).

These effects are due to the more rigid structure of *N,N'*-(1,4-phenylene)-bis(1,8-naphthalimide) (**NP2**) induced by the phenylene spacer in comparison to the flexible structures of other bis-naphthalimide derivatives. Additionally, the splitting of the symmetric stretching vibration band of carbonyl group of **NP2** is a result of nonequivalence of the carbonyl bonds which is caused by the slight deviation from planarity of the dicarboximide ring and compact packaging and interactions in the crystal state [37,38,39].

In general, the asymmetric and symmetric stretching vibration bands of the carbonyl groups of naphthalimide derivatives are shifted more than 50 cm^−1^ to lower frequencies in comparison to the phthalimide analogs [37,38,39]. For *N,N*-bis-(phthalimidopropyl)-*N*-propylamine the asymmetric stretching vibrations of carbonyl groups are split and lay at 1771 and 1764 cm^−1^, whereas the symmetric stretching vibrations of carbonyl groups appear at 1710 cm^−1^ [37]. Similarly, for *N,N*-bis-(phthalimido-propyl)-*N*-octylamine, where both the asymmetric and symmetric stretching vibration bands of the carbonyl groups are split, ν_as_C=O is observed at 1771 and 1764 cm^−1^, whereas ν_s_C=O lays at 1721 cm^−1^ and 1710 cm^−1^ [38]. The carbonyl bond and C-N bond lengths are 1.212 Ǻ and 1.394 Ǻ for *N,N*-bis-(phthalimidopropyl)-*N*-propylamine [37], and 1.210 Ǻ and 1.390 Ǻ for *N,N*-bis(phthalimidopropyl)-*N*-octylamine, respectively [38]. In the naphthalimide series, for *N,N*-bis-[3-(1,8-naphthalimido)propyl]-*N*-propylamine (**NP5**), asymmetric and symmetric stretching vibration bands of the carbonyl groups are observed at 1701 cm^−1^ and 1656 cm^−1^ (Table 1) and the carbonyl bond and C-N bond lengths are 1.223 Ǻ and 1.400 Ǻ, respectively [39]. These significant shifts of frequencies of the carbonyl groups are due to the increasing interaction between the carbonyl group and the nitrogen atom in naphthalimide derivatives in comparison to phthalimide derivatives, which reflects changes in the bond lengths of C=O and C-N, and as a consequence of this, increasing imide bond character [37]. 

**Figure 3:**
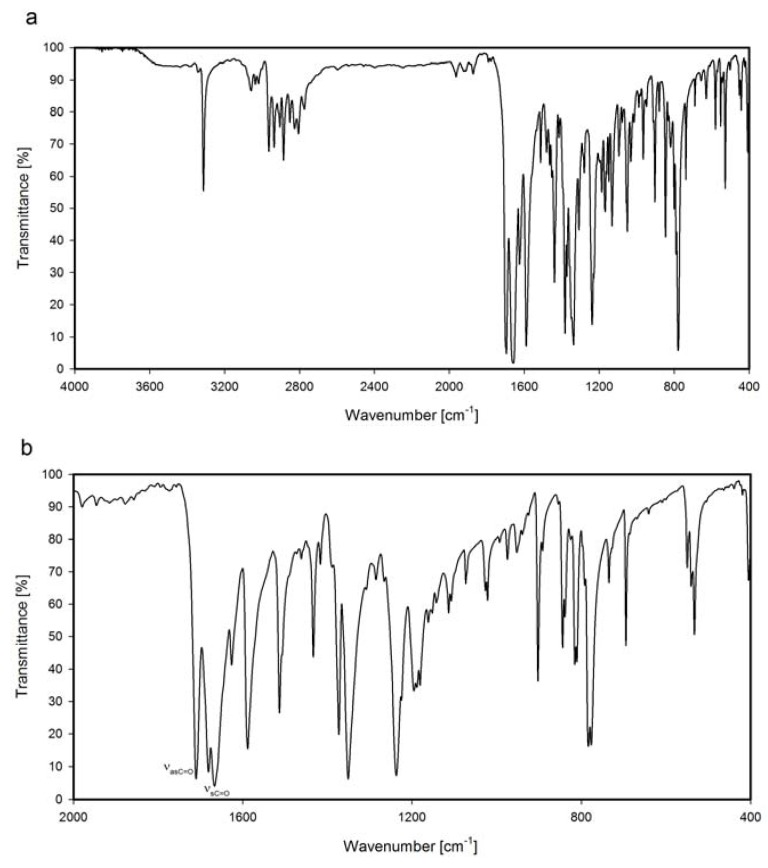
FTIR spectrum of *N,N*-bis[3-(1,8-naphthalimide)propyl]amine (**NP3**); (**a**) range 4000–400 cm^−1^ (**b**) range 2000–400 cm^−1^.

In the FTIR spectrum of *N,N*-bis[3-(1,8-naphthalimido)propyl]amine (**NP3**) an intense band of N-H stretching vibration at 3313 cm^−1^ is observed. (Figure 3, Table 1) The sharp, smooth shape of this band proves no interactions with any proton acceptors in **NP3**. In case of hydrochlorides, **NP6** and **NP7**, no “free” N-H band above 3000 cm^−1^ is present, and instead of this, very broad bands are observed with maxima at 2600 and 2460 cm^−1^, respectively. These vibration bands arise from hydrogen bond interactions, N^+^-H...Cl^−^.

**Figure 4:**
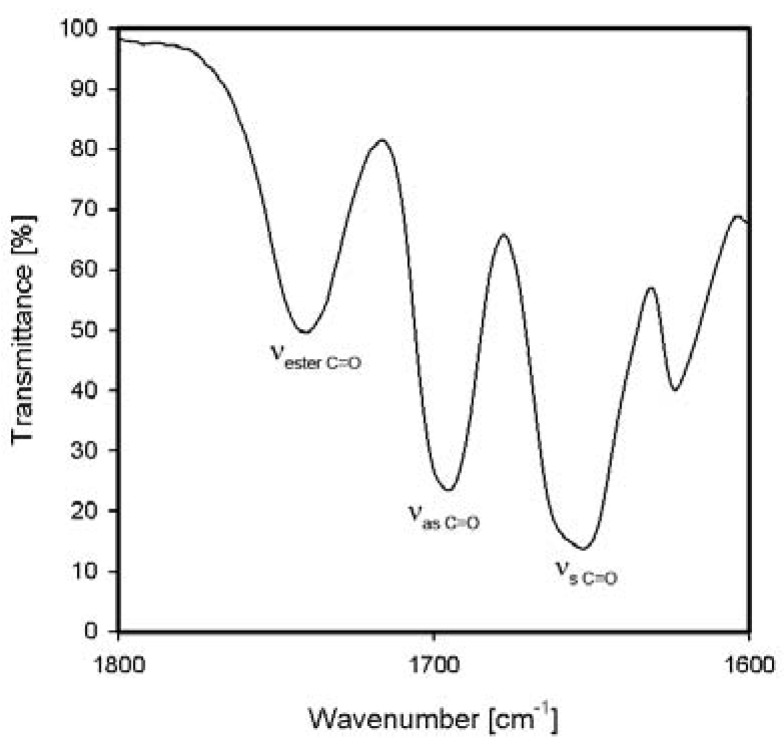
FTIR spectrum of carbonyl range of *N*-(2-ethoxy-2-oxoethyl)-*N,N*-bis[3-(1,8-naphthalimido)propyl]-*N*-propylammonium iodide (**NP8**).

For *N*-(2-ethoxy-2-oxoethyl)-*N,N*-bis[3-(1,8-naphthalimido)propyl]-*N*-propylammonium iodide (**NP8**), apart from bands of carbonyl groups of dicarboximide ring, new symmetric stretching vibration band of carbonyl group appears at 1741 cm^−1^ (Table 1, Figure 4). This band has no imide character and comes from carboethoxy substituent. 

### 2.3. ^13^C-NMR Spectra

^13^C-NMR chemical shifts for carbon atoms C-2, C-3, C-4 and carbonyl carbon atoms (C=O) of naphthalimide derivatives **NP2**–**NP9** at 298 K are given in Table 2. Because of the low solubility in organic solvents, the carbon spectra of *N,N'*-(1,4-phenylene)-bis(1,8-naphthalimide) (**NP2**), *N*-(2-etoxy-2-oxoethyl)-*N,N*-bis-[3-(1,8-naphthalimido)propyl]-*N*-propylammonium iodide (**NP8**) and *N*-ethyl-*N,N*-bis-[3-(1,8-naphthalimido)propyl]-*N*-propylammonium iodide (**NP9**) were measured in TFA-d. The remaining naphthalimide derivatives, **NP3**–**NP7**, were measured in CDCl_3_. The average value of the chemical shifts for C-2 of the naphthalimide derivatives **NP3**–**NP7** is 131.2 ppm, whereas the chemical shifts for C-3 and C-4 are 126.8 and 133.9 ppm, respectively. Chemical shifts of the carbonyl carbon atoms for naphthalimide derivatives **NP3**–**NP7** are observed at 164.1 ppm. Significant shifts towards higher values of ppm are encountered as expected for the ^13^C resonance frequencies of carbons C-2, C-3, C-4 and carbonyl carbon atoms for naphthalimide derivatives which were measured in TFA-d (Table 2). Kubo *et al.* showed that trifluoroacetic acid form strong hydrogen bonds O…H-O with *N*-substitued naphthalimide derivatives [40], changing the electron density in the naphthalimide ring. As a consequence of the lower electron density, ^13^C resonance frequencies shift toward higher chemical shifts. The very similar chemical shifts for naphthalimides with different *N*-substituents suggests that electronic effects of substituents on the electron distribution in the naphthalimide ring can be neglected.

**Table 2:** Selected ^13^C-NMR chemical shift of naphthalimide compounds in CDCl_3_.

Compound	Chemical ^13^C-NMR shift [ppm]			
	C-4	C-3	C-2	C=O
NP2 *	136.6	127.2	133.5	167.1
**NP3**	133.6	126.8	131.1	164.0
**NP4**	133.7	126.8	131.1	164.1
**NP5**	133.7	126.8	131.1	164.1
**NP6**	134.2	127.0	131.4	164.1
**NP7**	134.2	126.9	131.5	164.1
NP8 *	137.7	128.1	134.1	167.8
NP9 *	136.7	127.2	133.1	166.9

* TFA-d as solvent.

### 2.4. ^1^H-NMR Spectra

**Figure 5:**
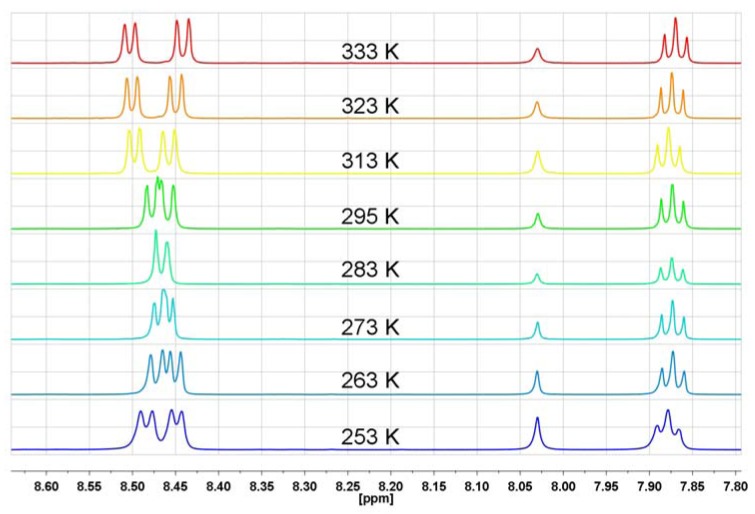
The range of aromatic protons of ^1^H-NMR spectrum in DMF-d_7_ for *N,N*-bis[3-(1,8-naphthalimido)propyl]-*N*-propylamine at different temperatures (**NP5**).

The ABM pattern of protons in *N*-substituted naphthalimide rings is expressed by one triplet and two doublets in the aromatic region of the ^1^H-NMR spectrum as for *N*-hydroxyethyl-1,8-naphthalimide, where the following signals are observed: 8.6 ppm (d, *J* = 6Hz, 2H), 8.22 ppm (d, *J* = 6Hz, 2H) and 7.8 ppm (t, *J* = 6 Hz, 2H) [41]. In the series of investigated bis-naphthalimides, *N,N*-bis[3-(1,8-naphthalimido)propyl]-*N*-propylamine (**NP5**) is characterized by the same ABM pattern, however only below 256 K or at temperatures higher than 300 K one triplet (H-3) and two doublets (H-2 and H-4) with *J* = 8 Hz are observed (Figure 5). In a wide range of temperature (253 K–333 K) the H-3 triplet shows practically no changes, whereas signals of H-2 and H-4 undergo an evolution from two doublets at 253 K, through two signals at 283 K, up to a double doublet at 333 K (Figure 5).

**Figure 6:**
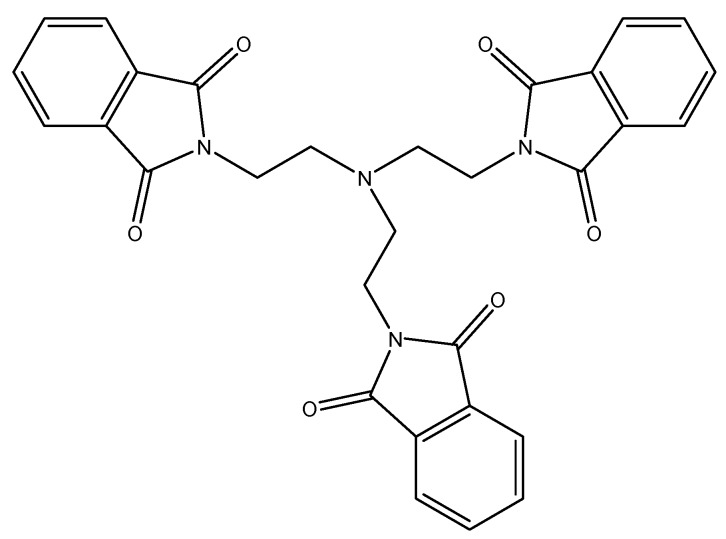
Structure of *N,N′,N′*′-(nitrilotriethylene)trisphthalimide.

A similar temperature behavior of the aromatic protons in ^1^H-NMR spectra was observed by Barrett for *N,N′,N′′*(nitrilotriethylene)trisphthalimide [35] (Figure 6).

**Figure 7:**
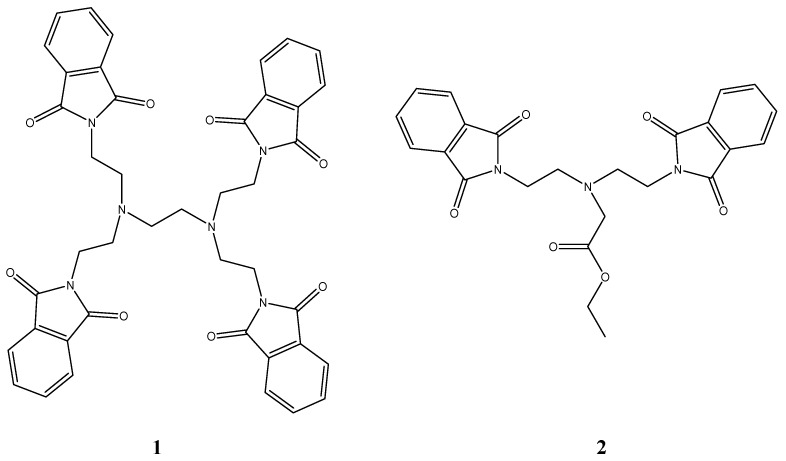
Structures of alkylaminephthalimides.

The proton NMR spectra of this compound in CDCl_3_ exhibited a spectacular temperature evolution from a sharp doublet of quartets at high temperature (323 K) to a complex second order behavior and then a singlet at 283 K. As the temperature was further lowered, a complex second order spectrum reappeared, followed by doublet of quartets at 213 K [35]. The unusual proton NMR characteristics of *N,N′,N′′*-(nitrilotriethylene)trisphthalimide have been explained by n-π interactions and rapid equilibration in solution of four assumed isomers. Using temperature dependency of chemical shifts, the enthalpy of interactions between π-electrons of phthalimide ring and n-electrons of amine was estimated to be ΔH^‡^ = 20 kJ/mol [35]. Some years ago Kahwa *et al.* carried out a very detailed study of crystal structures and ^1^H-NMR characteristics of some alkylaminephthalimides (Figure 7), including dendritic polyphthalimides [36].

They also observed a spectacular temperature evolution for the aromatic protons of compounds **1** and **2** in the ^1^H-NMR and claimed that the line profiles are attributable to n-π interactions. Like Barrett, those authors also calculated phthalimide-amine interaction enthalpy ΔH^‡^ for compound **2** to be 23 ± 1 kJ/mol in CD_3_CN, 17 ± 3 kJ/mol in CD_3_OD and 19 ± 5 kJ/mol in CDCl_3_.

Since bis-naphthalimide derivatives are structurally very similar to the compounds studied by Barrett [35] and Kahwa [36], we decided to check their hypothesis that n-π interactions were a responsible factor causing the unusual proton NMR characteristics. For our temperature-dependent ^1^H-NMR investigations we used *N,N*-bis[3-(1,8-naphthalimido)propyl]-*N*-propylamine hydrochloride (**NP6**), where no lone electrons pair exist on nitrogen atom.

**Figure 8:**
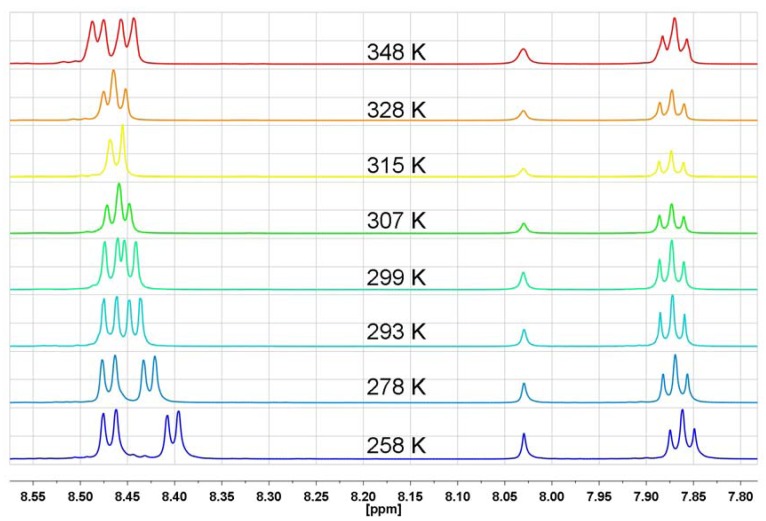
The range of aromatic protons of ^1^H-NMR spectrum in DMF-d_7_ for *N,N*-bis[3-(1,8-naphthalimido)propyl]-*N*-propylamine hydrochloride at different temperatures (**NP6**).

**Figure 9:**
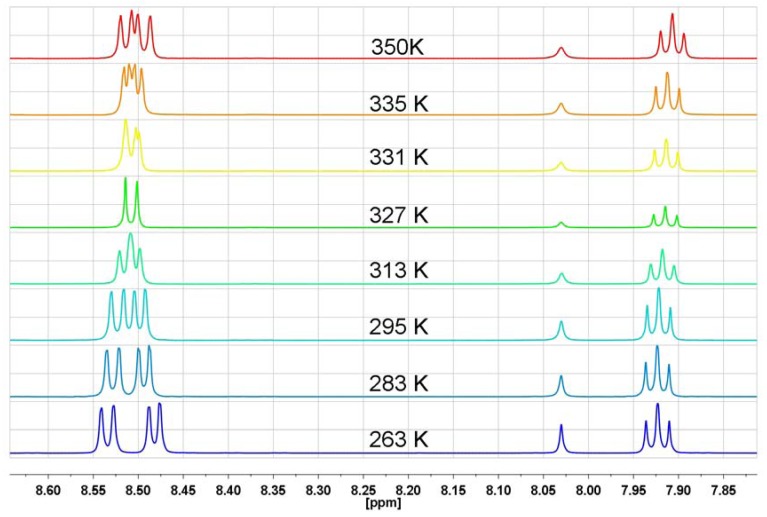
The range of aromatic protons of ^1^H-NMR spectrum in DMF-d_7_ for *N*-ethyl-*N,N*-bis[3-(1,8-naphthalimido)propyl]-*N*-propylammonium iodide (**NP9**) at different temperatures.

The ^1^H-NMR spectra of **NP6** taken in DMF-d_7_ in the temperature range 258 K–348 K reveal that the H-3 triplet is temperature independent and lays at 7.86 ppm, whereas the signals of H-2 and H-4 show unusual temperature behavior in the 8.37–8.48 ppm range. Starting as two distinct doublets at 258 K, as the temperature increases the signals evolve into two individual peaks at 315 K and again, as the temperature becomes higher the peaks undergo a transformation into two clear doublets at 348 K (Figure 8). The same temperature-dependent ^1^H-NMR behavior of *N,N*-bis[3-(1,8-naphthalimido)-propyl]-*N*-propylamine hydrochloride (**NP6**) and *N,N*-bis[3-(1,8-naphthalimido)propyl]-*N*-propyl-amine (**NP5**) excludes n-π interactions as a factor responsible for the unusual temperature proton behavior in the ^1^H-NMR spectra. *N,N*-bis[3-(1,8-naphthalimido)propyl]-*N*-propylamine hydrochloride (**NP6**) is a typical intermolecular complex with a N^+^-H…Cl^−^ hydrogen bond, where the proton is quickly moving between chloride anion and nitrogen. To avoid any suspicions that the proton transfer process is slower than the NMR time scale and n-electrons can even partially participate in n-π interactions, we measured the ^1^H-NMR spectra of a quaternary ammonium salt—*N*-ethyl-*N,N*-bis[3-(1,8-naphthalimido)propyl]-*N*-propylammonium iodide (**NP9**) in the 263 K–350 K temperature range in DMF-d_7_ (Figure 9).

In **NP9**, where four substituents are connected to the nitrogen atom, there is no doubt that n-electrons cannot interact with any π-electron system. As can be seen from Figure 9, the temperature-dependent ^1^H-NMR spectral feature of **NP9** with a quaternary nitrogen atom is very similar to that of **NP6** with a protonated nitrogen atom. The H-3 triplet is almost temperature independent, whereas the temperature evolution of the H-2 and H-4 signals is like that seen in **NP6**. In general, comparing the temperature-dependent ^1^H-NMR spectra of the amine **NP5**, protonated amine **NP6** and ammonium salt **NP9** one can state that the spectral features are very similar and there is no evidence for any participation of n-π interactions and rapid equilibration of naphthalimide isomers in solution. The only difference is that the “coalescence” temperature increase starts from 283 K for amine **NP5**, 315 K for protonated amine **NP6** and 327 K for the ammonium salt **NP9**.

**Figure 10:**
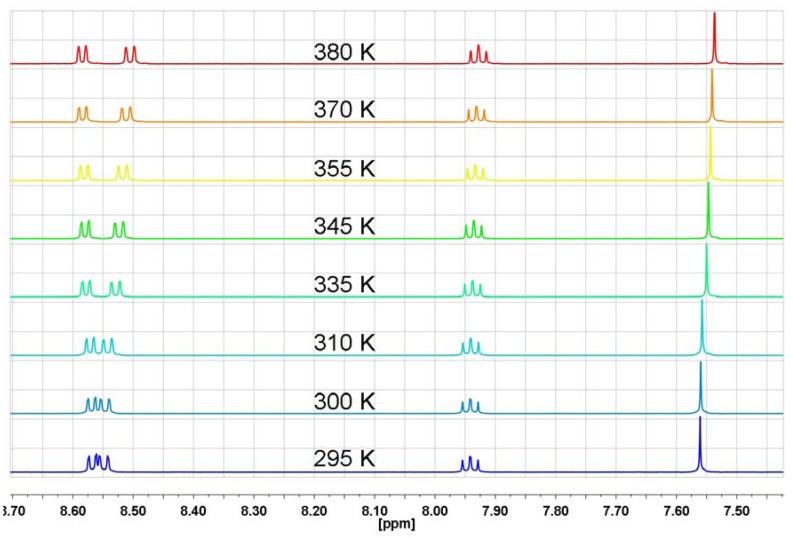
The range of aromatic protons of ^1^H-NMR spectrum in DMSO-d_6_ for *N,N′*-(1,4-phenylene)-bis(1,8-naphthalimide) (**NP2**) at different temperatures.

An additional proof of the absence of n-π interactions of bis-naphthalimide derivatives in solution is derived from the ^1^H-NMR temperature-dependent spectra of *N,N′*-(1,4-phenylene)-bis(1,8-naphthalimide) (**NP2**; Figure 10).

Because of the extremely low solubility of **NP2** in DMF-d_7_ the spectra were recorded in DMSO-d_6_ in which this derivative is slightly more soluble. The ^1^H-NMR temperature-dependent spectral behavior of *N,N′*-(1,4-phenylene)-bis(1,8-naphthalimide) (**NP2**), which does not contain a nitrogen atom in the substituent, is very similar to those previously observed. This means that n-π interactions play no fundamental role in the unusual temperature-dependent spectral features of naphthalimide derivatives.

**Figure 11:**
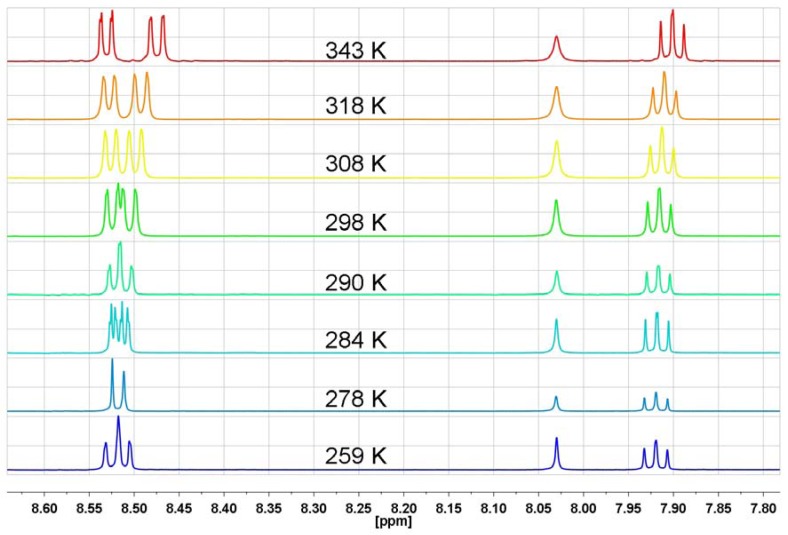
The range of aromatic protons of ^1^H-NMR spectrum in DMF-d_7_ for 1,8-naphthalimide at different temperatures.

Another factor which can cause an increase or decrease of proton shielding in naphthalimide rings and in turn could cause this unusual temperature ^1^H-NMR behavior is the geometry of 1,8-naphthalenedicarboximide derivatives. As Kovalevsky showed the naphthalenedicarboximide fragment can be formally divided into two 10π–electron naphthalene and the 7π electron dicarboximide fragments [42]. As a result the dicarboximide ring is more sensitive to the steric effects of substituents than to their electronic effects [42]. We have previously shown that both imide rings in *N,N*-bis[3-(1,8-naphthalimido)propyl]-*N*-propylamine (**NP5**) are non-planar [39]. This leads to deviations of the oxygen atoms from the imide average plane. It is very reasonable to suppose that these geometry deviations will vary with temperature and it can be a factor responsible for the unusual ^1^H-NMR behavior of the naphthalimide protons. To confirm this assumption we have measured ^1^H-NMR spectra of 1,8-naphthalimide over a wide range of temperatures in DMF-d_7_ (Figure 11).

As can be seen from Figure 11, the measured spectra of 1,8-naphthalimide have the same spectral features as the temperature-dependent spectra of the previously discussed bis-naphthalimide derivatives. The coalescence temperature is around 278 K, which is very close to the coalescence temperature of *N,N*-bis[3-(1,8-naphthalimido)propyl]-*N*-propylamine.

**Figure 12:**
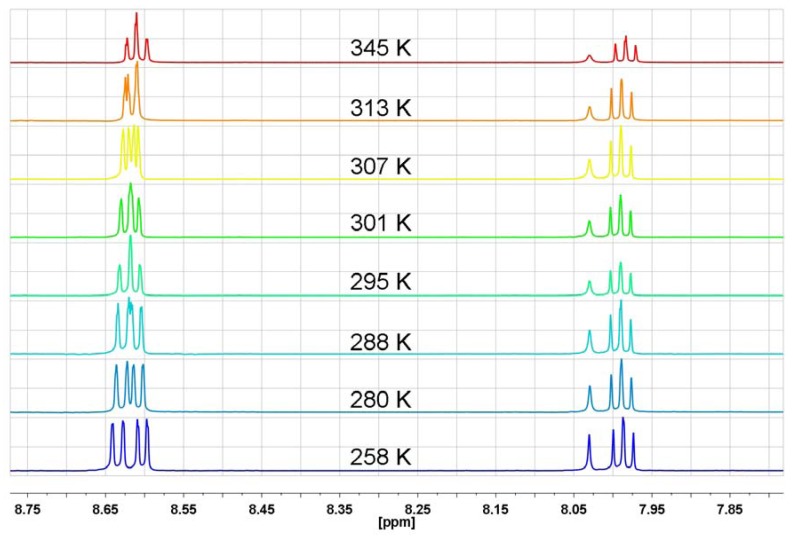
The aromatic proton range of the ^1^H-NMR spectrum in DMF-d_7_ for 1,8-naphthalenedicarboxylic anhydride at different temperatures.

To be sure that no electrons from the nitrogen atom are involved in this process we have also measured the temperature-dependent ^1^H-NMR spectra of 1,8-naphthalenedicarboxylic anhydride (**NP1**) under the same conditions. The obtained spectra reveal no significant changes in comparison to spectra of 1,8-naphthalimide, except for the coalescence temperature which is 295 K (Figure 12).

**Figure 13:**
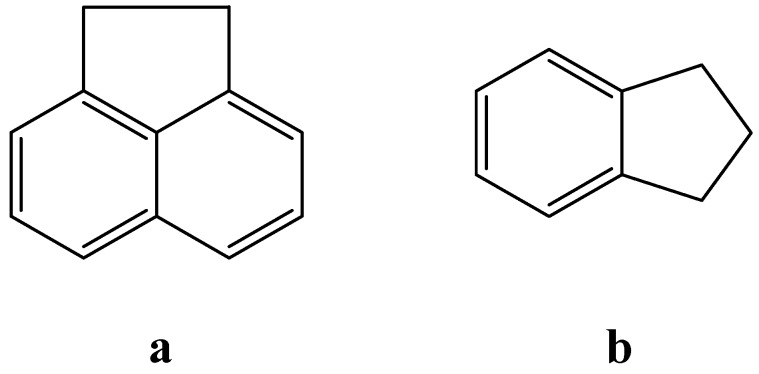
Structure of acenaphthene (**a**) and indane (**b**).

The above results clearly indicate that the temperature-driven changes of the geometry of the carbonyl group are the direct cause of the unusual behavior of the aromatic protons in the ^1^H-NMR spectra. To prove this suggestion the temperature-dependent ^1^H-NMR spectra of acenaphthene and indane were taken in CDCl_3_ and CD_3_OD respectively. The above compounds (Figure 13) are analogs of naphthalimide and phthalimide, but do not contain carbonyl groups.

**Figure 14:**
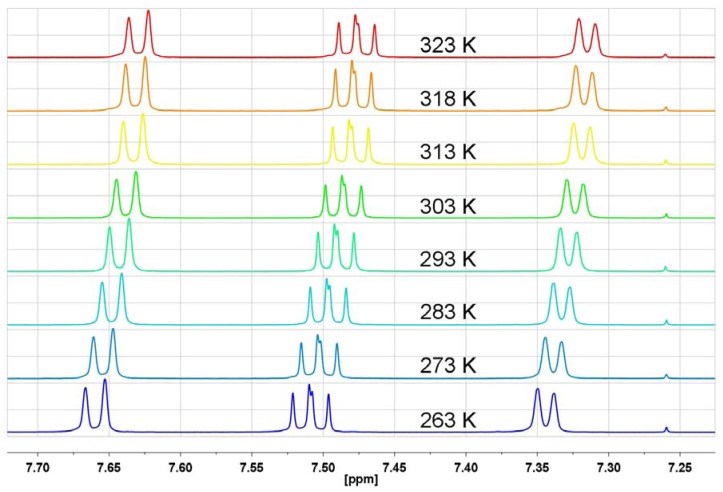
The aromatic proton region of the ^1^H-NMR spectrum in CDCl_3_ for acenaphthene at different temperatures.

**Figure 15:**
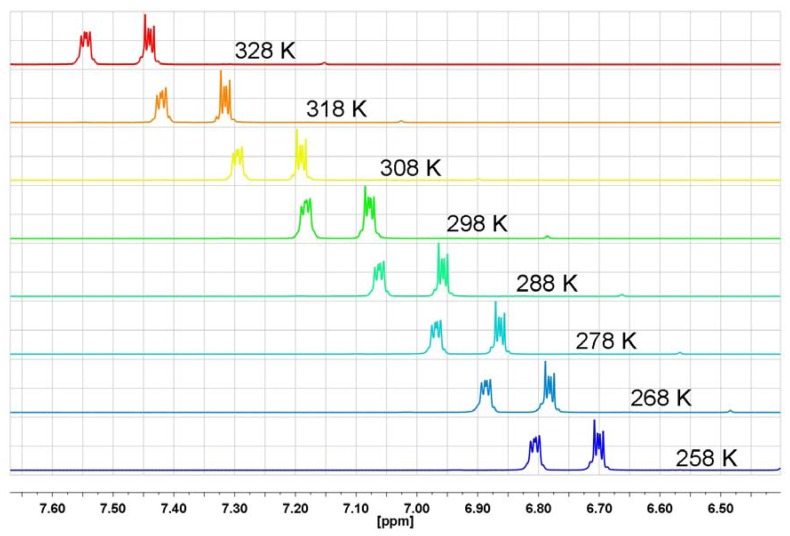
The aromatic proton region of the ^1^H-NMR spectrum in CD_3_OD for indane at different temperatures.

As can be seen from Figure 14 and Figure 15 for acenaphthene and indane, *i.e.*, compounds which do not contain carbonyl groups in the structure, there is no unusual aromatic proton behavior in the temperature-dependent ^1^H-NMR, except for regular temperature shifts of unchanged multiplets.

**Figure 16:**
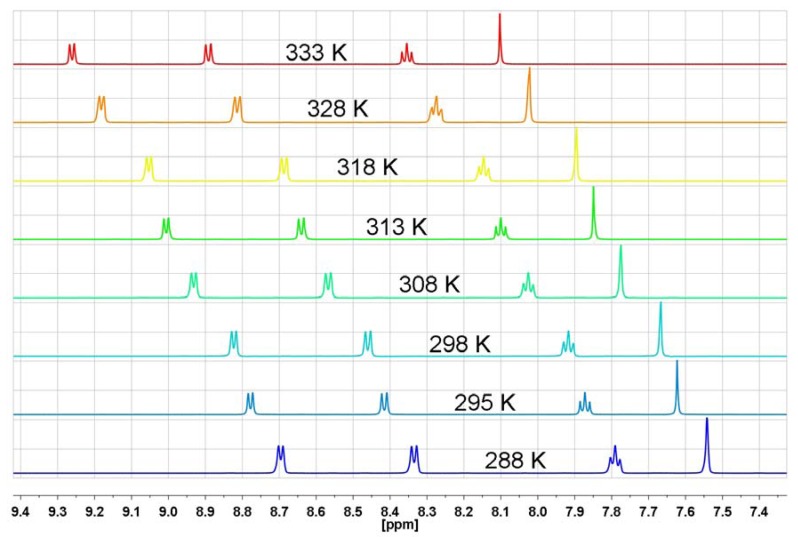
The aromatic protons region of the ^1^H-NMR spectrum in TFA-d for *N,N′*-(1,4-phenylene)-bis(1,8-naphthalimide) (**NP2**) at different temperatures.

Bis-naphthalimide derivatives are very sensitive to interactions with protic solvents, especially organic acids. Trifluoroacetic acid strongly interacts with the carbonyl groups of the dicarboximide ring, even at very low concentrations [40]. When the ratio of TFA-d to dicarboximide is very large, like in case where TFA-d is a solvent, the equilibrium constant between TFA-d and dicarboximide is shifted towards formation of a complex with strong intermolecular hydrogen bonds. It means that carbonyl group is caught in a cage of TFA-d and the flexibility of the carbonyl group will be very restricted. In such a case the rigid structure of the dicarboximide-TFA-d complex will be less sensitive to variable temperatures. The ^1^H-NMR spectra of *N,N′*-(1,4-phenylene)-bis(1,8-naphthalimide) (**NP2**) at different temperatures in TFA-d are shown in Figure 16.

Over a wide range of temperatures (288 K–333 K) no changes of the multiplicity of H-2, H-3 and H-4 occurred. This is a very strong additional evidence for the participation of carbonyl groups in the unusual temperature-dependent ^1^H-NMR characteristics of the naphthalimide protons. It also allows us to firmly reject the notion of n-π interactions as an explanation for the temperature behavior of the ^1^H-NMR of the aromatic protons of phthalimide and naphthalimide derivatives.

## 3. Experimental

### 3.1. General Procedures

The ^1^H-NMR spectra were recorded on a Bruker Avance DRX 600 MHz spectrometer over a wide range of temperatures. Typical conditions for the proton spectra were: pulse width 32°, acquisition time 5 s, FT size 32 K and digital resolution of 0.096 Hz per point. The ^13^C-NMR spectra were measured with a Varian Gemini 300VT spectrometer, operating at 75.4614 MHz. Typical conditions for the carbon spectra were: pulse width 60°, FT size 60 K and digital resolution of 0.6 Hz per point, the number of scans varied from 1,200 to 10,000 per spectrum. 2D-NMR spectra taken to correctly assign of the chemical shifts of protons and carbon atoms were recorded at 298 K on a Bruker Avance DRX 600 spectrometer operating at the frequencies of 600.315 MHz (^1^H) and 150.963 MHz (^13^C), and equipped with a 5 mm triple-resonance inverse probe head [1H/31P/BB] with a self-shielded *z* gradient coil (90° 1H pulse width 9.0 μs and 13C pulse width 13.3 μs). Chemical shifts are given in ppm versus TMS as internal standard. Chemical shifts for temperature ^1^H-NMR measurements are calculated versus chemical shift of solvent, assumed to be temperature independent. Infrared spectra were recorded in KBr pellets at 2 cm^−1^ resolution on a FT-IR Bruker IFS 66v/S instrument, evacuated to avoid water and CO_2_ absorption. Melting points were determined on a Melt-Temp II capillary melting point apparatus and are uncorrected. The deuterated solvents (>97% D), 1,8-naphthalenedicarboxylic anhydride, 1,8-naphthalimide, acenaphthene, and indane were purchased from Aldrich (Poznań, Poland) and used without any additional purification. Ethyl iodoacetate was obtained by reaction of commercially available ethyl chloroacetate with sodium iodide in acetone as a solvent.

### 3.2. Synthesis

*N,N′-(1,4-Phenyl)-bis-(1,8-naphthalimide)* (**NP2**). A mixture of 1,8-naphthalenedicarboxylic anhydride (4.05 g, 0.021 mol), *p*-phenylenediamine (1.08 g, 0.01 mol) and dimethylacetamide (25 mL) was refluxed under an argon atmosphere for 18 h. Then mixture was allowed to cool to room temperature and the brown crude product was filtered off and crystallized from DMSO. Yield: 1.54 g, as pale yellow crystals, mp > 360 °C. ^1^H-NMR (300 MHz, TFA-d): δ = 8.88 (d, 4H), δ = 8.52 (d, 4H), δ = 7.97 (t, 4H), δ = 7.72 (s, 4H); ^13^C-NMR (75 MHz, TFA-d): δ = 167.1, 136.6, 135.5, 133.5, 132.0, 130.0, 128.5, 127.2, 120.9; Anal. Calcd for C_30_H_16_N_2_O_4_ (468.46): C 76.92, H 3.44, 5.98 Found: C 76.62, H 3.65, N 5.94.

*N,N-bis-[3-(1,8-Naphthalimido)propyl]amine* (**NP3**). To a vigorously stirred mixture of 1,8-naphthalene-dicarboxylic anhydride (10.0 g, 50 mmol) in freshly distilled acetonitrile (100 mL) a solution of bis-(3-aminopropyl)amine (3.32 g, 25 mmol) in freshly distilled acetonitrile (20 mL) was added during 30 min. The mixture was stirred for 2 h at room temperature. The precipitated product was filtered off, washed with acetonitrile and dried under reduced pressure at 20 °C. The crude product was crystallized from nitromethane. Yield: 12.2 g (98%), mp 205–207 °C. ^1^H-NMR (300 MHz, CDCl_3_): δ = 8.57 (d, 4H), δ = 8.20 (d, 4H), δ = 7.73 (t, 4H), δ = 4.27 (t, 4H), δ = 2.73 (t, 4H), δ = 1.96 (m, 4H), δ = 1.84 (s, 1H); ^13^C-NMR (75 MHz, CDCl_3_): δ = 164.0, 133.6, 131.5, 131.1, 128.1, 126.8, 122.7, 47.2, 38.5, 28.6; Anal. Calcd for C_30_H_25_N_3_O_4_ (491.54): C 73.30, H 5.13, N 8.55 Found: C 72.76, H 5.28, N 8.55.

*N,N-bis-[3-(1,8-Naphthalimido)propyl]-N-dodecylamine* (**NP4**). A mixture of *N,N*-bis-[3-(1,8-naphthalimido)propyl]amine (5.0 g, 10.17 mmol), dodecyl bromide (4.0 g, 16.05 mmol), anhydrous Na_2_CO_3_ (0.59 g, 5.6 mmol) and dimethylformamide (60 mL) was heated and stirred at 100–120 °C for 12 h. After evaporation of solvent the oily residue was dissolved in chloroform and the precipitated inorganic salts were filtered off. The viscous product which was obtained after evaporation of chloroform, solidified after three months. Yield: 6.3 g (89%), mp 62–65 °C. ^1^H-NMR (300 MHz, CDCl_3_): δ = 8.56 (d, 4H), δ = 8.19 (d, 4H), δ = 7.73 (t, 4H), δ = 4.22 (t, 4H), δ = 2.63 (t, 4H), δ = 2.46 (t, 2H), δ = 1.90 (m, 4H), δ = 1.42 (m, 2H), δ = 1.23 (m, 18H), δ = 0.87 (t, 3H); ^13^C-NMR (75 MHz, CDCl_3_): δ = 164.1, 133.7, 131.5, 131.1, 128.1, 126.8, 122.8, 53.6, 51.5, 39.1, 31.9, 29.7, 29.4, 27.5, 27.0, 25.5, 22.7, 14.1; Anal. Calcd for C_42_H_49_N_3_O_4_ (659.86): C 76.45, H 7.48, N 6.37 Found: C 75.21, H 7.55, N 6.42 (hygroscopic product).

*N,N-bis-[3-(1,8-Naphthalimido)propyl]-N-propylamine* (**NP5**). We have reported previously another synthesis of this compound [39]. A mixture of *N,N*-bis-[3-(1,8-naphthalimido)propyl]amine (**NP4**, 5.0 g, 10.2 mmol), propyl bromide (1.88 g, 16.1 mmol), anhydrous Na_2_CO_3_ (0.59 g, 5.6 mmol) and dimethylformamide (60 mL) was heated and stirred at 100–120 °C for 10 h. Then, the mixture was cooled to 20 °C and solvent was evaporated under reduced pressure. The crude product was dissolved in chloroform and precipitated inorganic salts were filtered off. The solvent was evaporated and the pale-yellow residue was recrystallized twice from acetonitrile. Yield: 4.07 g (75%), mp 141–142 °C. ^1^H-NMR (300 MHz, CDCl_3_): δ = 8.56 (d, 4H), δ = 8.19 (d, 4H), δ = 7.75 (t, 4H), δ = 4.23 (t, 4H), δ = 2.63 (t, 4H), δ = 2.44 (t, 2H), δ = 1.90 (m, 4H), δ = 1.49 (m, 2H), δ = 0.90 (t, 3H); ^13^C-NMR (75 MHz, CDCl_3_): δ = 164.1, 133.7, 131.5, 131.1, 128.1, 126.8, 122.7, 55.5, 51.6, 39.1, 25.5, 20.2, 11.9; Anal. Calcd for C_33_H_31_N_3_O_4_ (533.62): C 74.28, H 5.86, N 7.87 Found: C 74.01, H 6.51, N 7.89.

*N,N-bis-[3-(1,8-Naphthalimido)propyl)]-N-propylamine hydrochloride* (**NP6**). *N,N*-bis-[3-(1,8-naphthalimido)propyl]-*N*-propylamine (0.26 g, 0.48 mmol) was dissolved in acetonitrile (50 mL) at 50 °C. To this solution, HCl (conc.) (75 mg) in acetonitrile (10 mL) was added dropwise with stirring and cooling. The mixture was evaporated under reduced pressure and solid product was dried in desiccator over P_4_O_10_. Yield: 0.27 g (99%), mp 111–130 °C (decomposition). ^1^H-NMR (300 MHz, CDCl_3_): δ = 12.03 (s, 1H), δ = 8.42 (t, 4H), δ = 7.75 (m, 4H), δ = 7.67 (t, 4H), δ = 4.24 (t, 4H), δ = 3.26 (t, 4H), δ = 3.02 (t, 2H), δ = 2.33 (m, 4H), δ = 1.89 (m, 2H), δ = 0.98 (t, 3H); ^13^C-NMR (75 MHz, CDCl_3_): δ = 164.1, 134.2, 131.5, 131.4, 128.0, 127.0, 122.1, 54.4, 50.4, 37.7, 22.5, 17.1, 11.2; Anal. Calcd for C_33_H_32_ClN_3_O_4_ 2.5H_2_O (615.12): C 64.44, H 6.06, N 6.83 Found: C 64.75, H 5.92, N 6.83.

*N,N-bis-[3-(1,8-Naphthalimido)propy)]-N-dodecylamine hydrochloride* (**NP7**). Solid *N,N*-bis-[3-(1,8-naphthalimido)propyl]-*N*-dodecylamine (1.0 g, 1.51 mmol) was dissolved in acetonitrile (100 mL) at 50 °C. To this solution, HCl (conc.) (0.3 g) in acetonitrile (10 mL) was added dropwise with stirring and cooling. The mixture was evaporated under reduced pressure and solid product was dried in desiccator over P_4_O_10_. Yield (1.04 g, 98%), mp. 188–189 °C. ^1^H-NMR (300 MHz, CDCl_3_): δ = 12.33 (s, 1H), δ = 8.42 (d, 4H), δ = 8.21 (d, 4H), δ = 7.73 (t, 4H), δ = 12.33 (s, 1H), δ = 4.27 (t, 4H), δ = 3.21 (t, 4H), δ = 3.00 (t, 2H), δ = 2.32 (m, 4H), δ = 1.79 (m, 2H), δ = 1.15–1.35 (m, 18H), δ = 0.88 (t, 3H); ^13^C-NMR (75 MHz, CDCl_3_): δ = 164.1, 134.2, 131.7, 131.5, 128.1, 126.9, 122.1, 52.6, 50.1, 37.6, 31.9, 29.6, 29.5, 29.4, 29.3, 28.9, 26.8, 23.2, 22.7, 22.3, 14.1; Anal. Calcd for C_42_H_50_ClN_3_O_4_ · 0.5H_2_O (705.32): C 71.52, H 7.29, N 5.96 Found: C 71.09, H 7.52, N 5.91.

*N-(2-Ethoxy-2-oxoethyl)-N,N-bis-[3-(1,8-naphthalimido)propyl]-N-propylammonium iodide* (**NP8**). A mixture of *N,N*-bis-[3-(1,8-naphthalimido)propyl]-*N*-propylamine (2.01 g, 3.77 mmol), an excess of freshly obtained ethyl iodoacetate (1.6 g, 7.50 mmol,) and acetonitrile (130 mL) was refluxed for 50 h. Then acetonitrile and excess of ethyl iodoacetate were removed under reduced pressure using a rotary evaporator. The crude product was crystallized twice from chloroform to give an orange to brown solid. Yield: 1.53 g (54%), mp 148–150 °C. ^1^H-NMR (300 MHz, TFA-d): δ = 8.72 (d, 4H), δ = 8.46 (d, 4H), δ = 7.92 (t, 4H), δ = 4.50 (t, 4H), δ = 4.33 (s, 2H), δ = 4.30 (q, 2H), δ = 3.97 (t, 4H), δ = 3.66 (t, 2H), δ = 2.53 (m, 4H), δ = 1.93 (m, 2H), δ = 1.33 (t, 3H), δ = 1.09 (t, 3H); ^13^C-NMR (75 MHz, TFA-d): δ = 166.9, 164.3, 136.7, 133.1, 131.8, 127.8, 127.2, 120.2, 64.0, 63.5, 58.5, 57.1, 37.5, 20.9, 15.5, 12.0, 8.6; Anal. Calcd for C_37_H_38_IN_3_O_6_ 3H_2_O (802.12): C 55.43, H 5.53, N 5.24 Found: C 55.66, 5.13, 5.47.

*N-Ethyl-N,N-bis-[3-(1,8-naphthalimido)propyl]-N-propylammonium iodide* (**NP9**). A suspension of *N,N*-bis-[3-(1,8-naphthalimido)propyl]-*N*-propylamine (4.45 g, 8.33 mmol) in excess iodoethane (15.0 g) was refluxed for 8 h, then cooled, filtered, dried and crystallized from acetonitrile/diethyl ether (1:1). Yield: 7.19 g (99%), mp 224–225 °C (decomposition)*.* ^1^H-NMR (300 MHz, TFA-d): δ = 8.73 (d, 4H), δ = 8.48 (d, 4H), δ = 7.94 (t, 4H), δ = 4.48 (t, 4H), δ = 3.63 (t, 4H), δ = 3.53 (q, 2H), δ = 3.34 (t, 2H), δ = 2.46 (m, 4H), δ = 1.90 (m, 2H), δ = 1.50 (t, 3H), δ = 1.12 (t, 3H); ^13^C-NMR (75 MHz, TFA-d): δ = 167.8, 137.7, 134.1, 132.7, 128.7, 128.1, 121.0, 61.5, 57.0, 55.8, 38.5, 21.4, 15.9, 9.5, 7.0; Anal. Calcd for C_35_H_36_IN_3_O_4_ 0.5H_2_O (698.59): C 60.17, H 5.34, N 6.01 Found: C 59.96, H 5.30, N 6.01.

## 4. Conclusions

New bis-naphthalimide derivatives containing functionalized spacers were obtained using 1,8-naphthalenodicarboxylic anhydride as a starting material. Yields of the reactions in most cases were over 75% in high boiling solvents. The obtained bis-naphthalimide derivatives are very stable. Most of the synthesized naphthalimide derivatives are barely soluble in water, however, in the presence of solubilizers, like gemini surfactants with high HLB value, their solubilities are satisfactory. The synthesized bis-naphthalimide derivatives have been studied by FTIR in the solid state and ^1^H and ^13^C-NMR in solution. The infrared spectra revealed some nonequivalency of the carbonyl groups in the solid state caused by the crystal structure. The carbonyl groups are very sensitive to polar protic organic solvents and formation of intermolecular hydrogen bonds between the carbonyl groups and trifluoroacetic acid has been shown by ^13^C-NMR. A detailed ^1^H-NMR study of the bis-naphthalimide derivatives at different temperatures demonstrated the unusual behavior of the aromatic protons of the naphthalimide rings. We proved that changing geometry of the carbonyl groups is responsible for this temperature behaviour of the aromatic protons of naphthalimide derivatives and n-π interactions play no role in the unusual temperature features of the ^1^H-NMR spectra.
